# Lowering Etoposide Doses Shifts Cell Demise From Caspase-Dependent to Differentiation and Caspase-3-Independent Apoptosis via DNA Damage Response, Inducing AML Culture Extinction

**DOI:** 10.3389/fphar.2018.01307

**Published:** 2018-11-13

**Authors:** Emanuele Bruni, Albrecht Reichle, Manuel Scimeca, Elena Bonanno, Lina Ghibelli

**Affiliations:** ^1^Department of Biology, University of Rome “Tor Vergata,”, Rome, Italy; ^2^Department of Internal Medicine III, Haematology and Oncology, University Hospital of Regensburg, Regensburg, Germany; ^3^Department of Biomedicine and Prevention, University of Rome “Tor Vergata”, Rome, Italy; ^4^Department of Experimental Medicine, University of Rome Tor Vergata, Rome, Italy; ^5^Diagnostica Medica and Villa dei Platani, Avellino, Italy

**Keywords:** apoptosis, caspase-3, caspase-2, metronomic chemotherapy, AML, differentiation

## Abstract

Cytotoxic chemotherapy, still the most widely adopted anticancer treatment, aims at eliminating cancer cells inducing apoptosis with DNA damaging agents, exploiting the differential replication rate of cancer vs. normal cells; efficiency is evaluated in terms of extent of induced apoptosis, which depends on the individual cell sensitivity to a given drug, and on the dose. In this *in vitro* study, we report that the concentration of etoposide, a topoisomerase II poison widely used in clinics, determines both the kinetics of cell death, and the type of apoptosis induced. We observed that on a set of myeloid leukemia cell lines, etoposide at high (50 uM) dose promoted a rapid caspase-3-mediated apoptosis, whereas at low (0.5 uM) dose, it induced morphological and functional granulocytic differentiation and caspase-2-dependent, but caspase-3-independent, cell death, displaying features consistent with apoptosis. Both differentiation and caspase-2- (but not 3)-mediated apoptosis were contrasted by caffeine, a well-known inhibitor of the cellular DNA damage response (DDR), which maintained cell viability and cycling, indicating that the effects of low etoposide dose are not the immediate consequence of damage, but the result of a signaling pathway. DDR may be thus the mediator responsible for translating a mere dosage-effect into different signal transduction pathways, highlighting a strategic action in regulating timing and mode of cell death according to the severity of induced damage. The evidence of different molecular pathways induced by high vs. low drug doses may possibly contribute to explain the different effects of cytotoxic vs. metronomic therapy, the latter achieving durable clinical responses by treating cancer patients with stable, low doses of otherwise canonical cytotoxic drugs; intriguingly caspase-3, a major promoter of wounded tissue regeneration, is also a key factor of post-therapy cancer repopulation. All this suggests that cancer control in response to cytotoxic drugs arises from complex reprogramming mechanisms in tumor tissue, recently described as anakoinosis.

## Introduction

Induction of apoptosis with DNA damaging drugs is still the most used anticancer chemotherapeutic approach for many types of human cancers. Modern cytotoxic protocols generally guarantee response, implying efficient cancer cell killing and tumor size reduction. However, with few exceptions, cancer cells surviving the treatment, repopulate the depleted tumor tissue with more aggressive phenotypes, leading to relapse, and patients’ death. The mechanisms promoting repopulation include immune infiltrations and modulation by stroma. Stroma provides tissue autonomous and systemic support for regenerating ‘wounded’ cancer tissue. These considerations led to addressing stroma or immune cells as anticancer therapy (e.g., immunotherapy), counteracting tumor repopulation.

A vast pre-clinical literature describes in detail the cellular consequences of DNA damage. Damage response goes beyond the mere induction of apoptosis, rather encompassing multiple cell-autonomous and cell-non-autonomous processes through complex signal transduction pathways, being potential targets for novel adjuvant protocols following cytotoxic therapies.

A key cell response to DNA insults consists in a complex phosphorylation cascade (the DNA damage response, DDR) organized by Ataxia telangectasia mutated (ATM) kinase, which coordinates cell response to the damage, blocking cell cycle and organizing DNA repair (Caputo et al., 2012). DDR also elicits a plethora of pro-survival, pro-differentiation, pro-senescence (Sherman et al., 2011) and pro-apoptotic signals (including caspase-dependent (Pabla et al., 2007) and -independent (Agarwal et al., 2006) apoptosis), determining the way injured cells cope with stress conditions. Therefore, DDR acts as a key regulator of injured cell fate, pondering the effects that DNA damage exerts on cells, and deciding whether allowing damaged cell survival, implying risk of mutation and possibly cancer initiation, or promoting cell demise with risk of tissue degeneration or fibrosis.

Importantly, DDR coordinates not only cell, but also tissue dynamics, being an important non-cell-autonomous regulatory hub. Key DDR actors such as p53, Chk1, and Chk2 modulate signals regulating cell-to-cell communication (Raulet and Guerra, 2009); notably, DDR is emerging as an intriguing link with the immune response elicited by injured tissues (Nakad and Schumacher, 2016), establishing multiple correlations between DNA damage, innate immunity and cancer (Pateras et al., 2015). Moreover, DDR may favor or contrast PGE2-mediated repopulation (Allen et al., 2015), suggesting a complex interaction between the phoenix rising pathway and DDR, which may lead to different effects depending on the tissue context. Therefore, according to the situation present in a specific microenvironment, DDR can act as a pro- or anti-tumoral pathway (Agarwal et al., 2006; Pabla et al., 2007); consequently, addressing DDR toward an anti-tumor asset may be a novel goal of anticancer therapies.

Apoptosis, though stably eliminating tumor cells, thus fulfilling the primary goal of anticancer therapies, may nonetheless act as Trojan horse, since caspase-3, the main executioner of the intrinsic and extrinsic apoptotic pathways (Coppola and Ghibelli, 2000), is emerging as a major actor of tumor tissue reconstitution after cytotoxic therapy (Huang et al., 2011; Shalini et al., 2014). Caspase-3 proteolytically activates cytosolic phospholipase A2, which in turn activates cyclooxygenase-2 to produce prostaglandin E2 (Kurtova et al., 2014), a mediator promoting proliferation of surviving cancer stem cells through paracrine signaling, leading to repopulation. This behavior is reminiscent of the regular post-wound regeneration of normal tissues, where caspase-3 acts as a “counter” of cell loss, contributing to the replacement of a proper number of cells in the injured organ (the phoenix rising pathway (Li et al., 2010). In cancer, repopulation is accompanied by increased malignancy (Flanagan et al., 2016), and clinically coincides with post-therapy tumor relapses. Notably, not all types of apoptosis depend on caspase-3, thus opening the way to a selection of agents promoting caspase-3-independent apoptosis in therapeutic settings. All this information indicates that not only the extent of therapy-induced cell loss, but also the mode of cell death, is important to determine success of a cytotoxic therapy, because apoptotic cells, before collapsing, emit communicative signals determining the fate of the treated cancer tissue as a whole.

In this study, we report that the occurrence of apoptosis through caspase-3-mediated or -independent mechanisms, depends on the concentration of the drug used, low etoposide doses eliciting a DDR that can drive injured AML cells to caspase-3-independent apoptosis and granulocytic differentiation.

## Materials and Methods

### Cell Cultures

U937 (human tumor monocytes) were cultured in RPMI 1640 medium (Euroclone), supplemented with 10% fetal calf serum (FCS; Gibco), 2 mM L-glutamine, 100 IU/mL penicillin and streptomycin (Euroclone), and kept at 37 °C in a humidified atmosphere of 5% CO_2_ in air. All experiments were performed on cells in the logarithmic phase of growth under condition of > 98% viability (as determined by trypan blue exclusion). In each experiment cells were kept at the density of 10^6^ cells/mL.

### Treatments

Etoposide (Sigma-Aldrich), was used at different concentrations as indicated, and kept throughout the experiments. Caffeine (Sigma-Aldrich, 1 mM) was used as inhibitor of ATM-mediated DNA damage response (Caputo et al., 2012) and added together with etoposide. Phorbol 12-myristate 13-acetate (PMA, 200 ng/mL) was used as degranulating agent (Parmley et al., 1983) or as stimulator of oxidative burst (Collins et al., 1979), and kept for 30 min before either measurement. Z-DEVD-fmk (50 uM) was used to inhibit caspase-3; Z-VDVAD-fmk (2 uM) as caspase-2 inhibitor; Z-VAD-fmk (10 uM) as pan caspase-inhibitor. Each inhibitor was added 1 h before the apoptotic inducer.

### Cell Cycle Analysis

Cell cycle was analyzed by evaluating intracellular DNA content by incorporation of the stoichiometric propidium iodide (PI) dye, and assessing fluorescence as cell distribution by flow cytometry. For the measurements, 106 cells were washed twice in PBS, fixed in ethanol/PBS (3:1), kept for 24 h at -20°C, washed twice, treated with 100 ug/mL RNAs and 10 ug/ml PI at 37°C for 15 min in the dark. Samples were then analyzed by flow cytometry. The fraction of cells showing < 2n DNA content is defined as the sub-G1 region and include apoptotic cells.

### Apoptotic Nuclear Fragmentation

The fraction of cells presenting apoptotic nuclei, among the total cell population was calculated by counting at the fluorescence microscope at least 300 cells in at least three different, randomly selected microscopic fields, after staining cells with the cell-permeable DNA-specific dye Hoechst 33342 directly added to the culture medium at the final concentration of 10 ug/mL (Ghibelli et al., 1995; Dini et al., 1996).

### Analysis of Superoxide and PMA-Mediated Effects

Superoxides were analyzed using dihydroethidium (DHE, excitation 370 nm/emission superoxide: 5 uM DHE was added directly to the cell samples and incubated at 37°C in the dark for 30 min; then cells were analyzed by flow cytometer. PMA-induced superoxides and degranulation: 10^6^ cells were incubated with 200 ng/mL PMA and 5 uM DHE in PBS for 30 min at 37°C in the dark; then cells were analyzed by flow cytometer for DHE staining and FSC/SSC.

### Flow Cytometry

Flow cytometric analyses were performed with FACScalibur (Becton Dickinson) equipped with a 488 nm laser. Data were recorded and analyzed with WinMdi 2.9 software. The analyses were performed by counting 20,000 cells/treatment.

### TEM Analysis

Cells were fixed in 4% paraformaldehyde, post-fixed in 2% osmium tetroxide and embedded in EPON resin for morphological studies. After washing with 0.1M phosphate buffer, the sample was dehydrated by a series of incubations in 30, 50, and 70, ethanol (Scimeca et al., 2014). Dehydration was continued by incubation steps in 95% ethanol, absolute ethanol, and hydropropyl methacrylate; then samples were embedded in Epon (Agar Scientific, Stansted Essex CM24 8GF United Kingdom). After incubation, cells were cut and stained with heavy metals solutions as described (Reynold, 1963).

### Western Blotting Analysis

Whole cell extracts (5 × 106 cells) were prepared after lysis in hypotonic buffer (10 mM NaCl, 3 mM MgCl2, 10 mM *Tris-HCl* pH 7.5, 0.1% SDS, 0.1% Triton, 0.5 mM EDTA; protease inhibitor cocktail (Sigma-Aldrich) and 1 mM DTT (Sigma-Aldrich) were added just before use). For western blot analysis, equal amounts of proteins (20 ug) of total cell extracts were separated by using a sodium dodecyl sulfate-(SDS) polyacrylamide gel (SDS–PAGE; 10% acrylamide separating gel, 4% acrylamide stacking gel) after denaturing samples by boiling (according to the method of Laemmli). After electrophoresis, proteins were transferred to PVDF membranes (GE Healthcare Life Sciences). Membranes were blocked in PBS-Tween (0.1%) with 5% of dry milk for 1 h at room temperature or at +4°C overnight. The membrane was washed with PBS-Tween and incubated with the following primary antibodies diluted in PBS-Tween with 5% dry milk or in PBS-Tween with 5% BSA (for caspase-8): mouse anti-caspase-3 (Santa Cruz Biotechnology), mouse anti-caspase-6, mouse anti-caspase-8 and rabbit anti-caspase-9 (Cell Signal), mouse anti-caspase-2 (Cell Signal), used in the dilutions of 1:1000. The washing step was repeated with PBS-Tween and the membrane was incubated with specific HRP-conjugated secondary antibodies (Santa Cruz Biotechnology), with the following dilutions: 1:4000 for anti-caspase-3, -2, -6, and -8, 1:5000 for anti-caspase-9. After washing with PBS-Tween, the signals of specific immunoreactive proteins were visualized using the Amersham ECL Plus Western Blotting Detection System Kit (GE Healthcare Life Sciences) and the Image Quant LAS 4000 mini (GE Healthcare Life Sciences).

### Statistical Analysis

Statistical analysis was performed using Student’s *t*-test and *P*-values < 0.05 were considered significant. Data are presented as mean ± SD.

## Results

### 0.5 uM Etoposide Induces Atypical, Caspase-Independent Apoptosis Through Caspase 2 Activation

50 and 0.5 uM etoposide totally eliminate U937 cells, though with different kinetics, reaching culture extinction at 24 h vs. 72 h, respectively. The process occurs by apoptosis in both cases, inducing apoptotic features such as: nuclear vesiculation; loss of mitochondrial trans-membrane potential; DNA digestion (sub-G1 peak, see below); cytochrome c release and Bax translocation to mitochondria; glutathione loss (data not shown).

**FIGURE 1:**
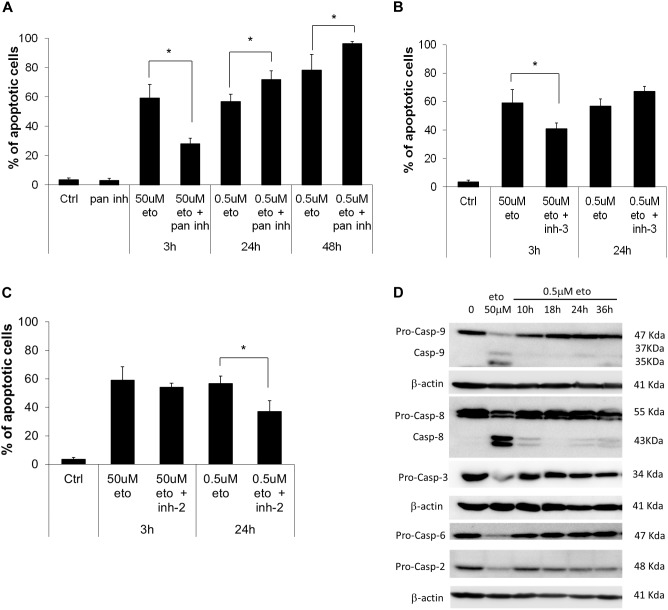
0.5 uM etoposide induces caspase-independent apoptosis. **(A)** Fraction of apoptotic U937 cells after treatment with 50 uM or 0.5 uM etoposide ± pan caspase inhibitor (Z-VAD-fmk). Effect of caspase-3 **(B)** and caspase-2 **(C)** inhibitors on 50 uM and 0.5 uM etoposide-induced apoptosis (measured as nuclear fragmentation). Results are the average of 3 independent measurements ± SD: ^∗^*p* < 0.05. **(D)** Caspase activation evaluated by western blotting analysis at the indicated times of etoposide (50 uM and 0.5 uM) treatments. Results are representative of 2 independent experiments.

We explored the role of caspases in the apoptosis induced by the two treatments; we found that the pan-caspase inhibitor, which inhibits all the apoptosis-related caspases but caspase-2 (Shi et al., 2009), strongly reduced apoptosis induced by 50 uM apoptosis; instead, upon 0.5 uM etoposide, the inhibitor not only did not prevent apoptosis, but even, apoptosis was significantly increased (Figure 1A). We specifically analyzed the effect of caspase-3 inhibitor, showing that while it strongly prevents 50 uM etoposide-induced apoptosis, it does not reduce (in fact, increases) apoptosis induced by the low dose (Figure 1B). In the latter case, this implies that (a), cells developed apoptosis without the intervention of the canonical caspases and especially caspase-3 and (b) caspases may exert a paradoxical anti-apoptotic role.

An opposite situation instead was found for caspase-2: the specific inhibitor did not affect apoptosis induced by 50 uM etoposide, but strongly inhibited 0.5 uM-induced apoptosis (Figure 1C), showing that caspase-2 is responsible for 0.5 uM etoposide-induced apoptosis. This is in line with studies reporting that caspase-2 is activated by DNA damage as an apical caspase (Sidi et al., 2008) and mediates the consequent cell responses, including apoptosis. Instead, apoptosis inducing factor (AIF), which is often responsible for DNA and protein cell dismantling in DNA damage-induced apoptosis, does not play any role in 0.5 uM etoposide-induced apoptosis, since it does not leave its steady-state mitochondria localization (data not shown).

Figure 1D shows the activation state of the caspases involved; they are strongly proteolytically activated by 50 uM etoposide, but activation occurs also at the low dose, albeit to a much lower extent. The inhibitors experiments show that this latter activity does not have an apoptotic meaning, rather, it may contrast or delay apoptosis, putting in context the paradoxical pro-apoptotic role of all the caspase inhibitor tested (Figure 1B; De Nicola et al., 2017). The only caspase strongly activated by 0.5 uM etoposide is caspase 2, confirming its apical role, as pointed out by the inhibition experiments (Figure 1C). Caspase 2 activation is even stronger upon 50uM etoposide, even though it does not play an apical role in that case.

**FIGURE 2:**
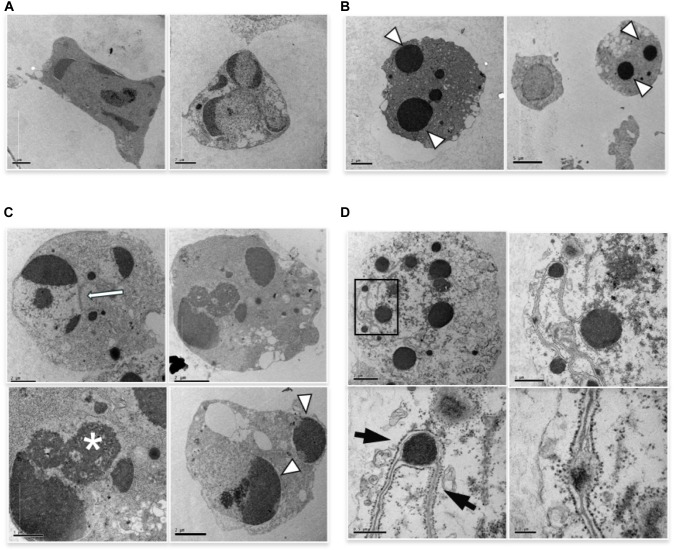
TEM analysis of cells induced to apoptosis by etoposide. **(A)** U937 treated with 50 uM etoposide showing canonical apoptotic figures, including chromatin condensation at the nuclear margin. **(B–D)** Cells treated with 0.5 uM etoposide. **(B)** shows recognizable apoptotic figures, but with atypical membrane features, e.g., two nuclear fragments juxtaposed and separated by pore-enriched double nuclear membrane (white arrow) or interruptions of the nuclear membrane (arrowhead). ^∗^ indicates nucleolar alterations. **(C)** Condensed chromatin spherical masses without nuclear membrane (arrowhead). **(D)** Fourfold membrane network connecting dense chromatin masses in late apoptosis, with the outer sheet wrapped by ribosomes (arrows).

We performed ultrastructural analysis to appreciate an eventual difference in apoptotic morphology of the two forms of apoptosis. TEM analysis of cells induced to apoptosis by high vs. low etoposide doses, reveals slight but consistent morphological differences. At 50 uM, we observed chromatin condensation at the nuclear margin and nuclear vesiculation with intact nuclear membrane, with morphologies consistent with the budding or cleavage types of nuclear fragmentation, previously described in U937 cells (Dini et al., 1996; De Nicola et al., 2006a,b; Figure 2A). The 0.5 uM dose induces *bona fide* apoptotic features, with typical nuclear vesiculation, which however, presents peculiar defects on the nuclear membranes (arrows and arrowheads in Figure 2B). Atypical morphologies are also detectable, such as the chromatin spherical masses with no or interrupted membrane, as shown in Figure 2C. Other peculiar features are presented in Figure 2D, including dense chromatin granules surrounded by a four-fold membrane, which connects other similar granules throughout the cells: this occurs in cells presenting a cytosol with poorly organized texture, suggesting a late apoptotic stage.

The images in Figure 2C, showing apoptotic cells with spherical, membrane-free, highly condensed chromatin masses are compatible with chromosome-to-apoptotic body transition, possibly being sign of mitotic catastrophe, event that often accompanies damage-induced cell death.

In conclusion, 0.5 uM etoposide induces apoptotic cell death, without signs of necrosis or autophagy, via caspase-2, but without caspase-3 intervention.

### 0.5 uM Etoposide Induces Nuclear Blebbing and Granulocytic Differentiation

**FIGURE 3:**
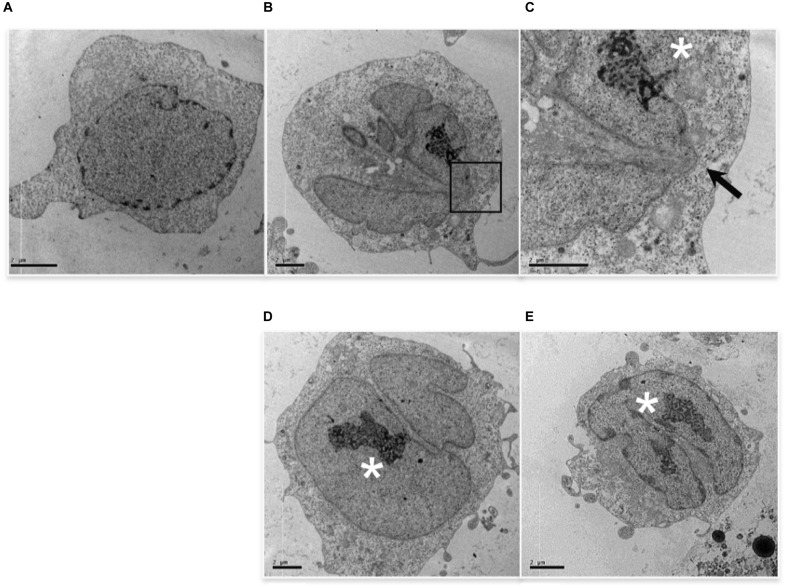
0.5 uM etoposide induces nuclear blebbing. Untreated U937 cells **(A)** and cells treated with 0.5 uM etoposide **(B–E)**. **(C)** is the magnification of the area indicated by the square in **(B)**. ^∗^ marks nucleoli in transition from ring-shaped nucleolus and nucleolus with nucleolonemas. Results are representative of 2 independent experiments.

TEM analysis showed additional alterations caused by low etoposide doses. 0.5 uM (but not 50 uM) etoposide produces at high frequency nuclear blebbing, with paroxysmal invagination of the double nuclear membrane within the nuclear sap, as shown in Figure 3. This is accompanied by proteolysis of nuclear lamin A in a caspase-independent manner (study in progress).

Cells with nuclear blebbing present normal cytosol and organelles, and normally diffused chromatin, but atypical nucleoli, which acquire a characteristic organization (Figure 3), consisting of both nucleolus in transition from ring-shaped nucleolus and nucleolus with nucleolonemas, and grossly shaped nucleolus with prominent nucleolonemas containing mainly dense granular elements (Figure 3). The nucleolonema is a fundamental substructure of the nucleolus, and its skeleton is the tandem arrangement of the fibrillary components, which are resting harbors or storage of ribosomal DNA; therefore, these nucleolar alterations, associated to nuclear blebbing, might indicate an increased synthesis of ribosome components, a feature that is usually associated with gene expression reprogramming. Intriguingly, it is reported that DNA damage activates nucleolar caspase-2 as apical event (O’Byrne and Richard, 2017), causing alterations in nucleolar proteins localization (Ando et al., 2017). Since caspase-2 is responsible for 0.5 uM-induced apoptosis (Figure 1C), it is tempting to associate DNA-damage caspase-2 activation with nuclear morphological alterations.

**FIGURE 4:**
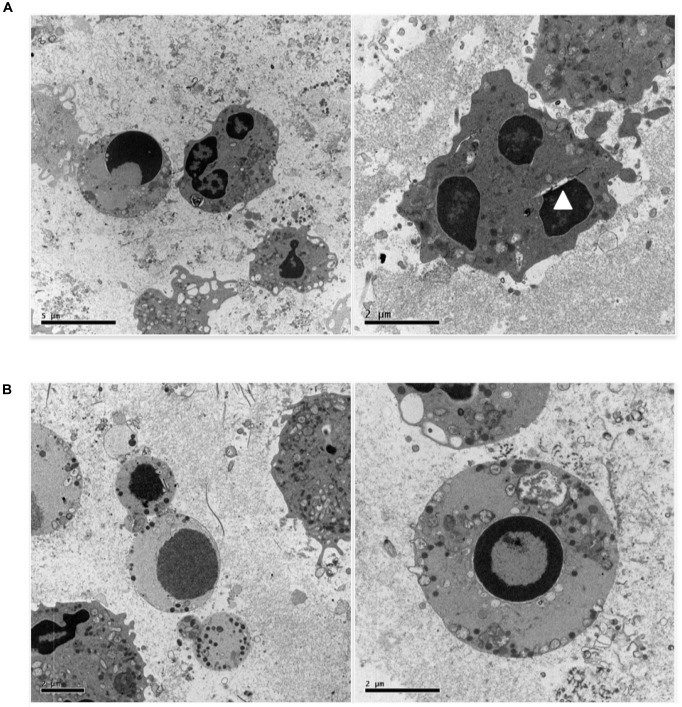
0.5 uM etoposide promotes granulocytic-like morphology and differentiation-related apoptosis. **(A)** Cells with peculiar fragmentation of the nuclei in 2–3 segments with irregular shape, strongly resembling polymorphonuclear cell nuclei. Nuclei show dilated perinuclear membrane blank (arrowhead). **(B)** Cells presenting apoptotic nuclei and granulocyte-like characteristics, including electrondense granules at the cell periphery or in the process of being extruded.

Figure 4A shows a further set of pictures of 0.5 uM etoposide-treated U937 cells, whose main feature is the peculiar fragmentation of nuclei in 2–3 segments with irregular shape; the nuclear membrane is hardly detectable, but a gap between the nuclear margin and the cytosol is often visible (arrowhead). The chromatin is condensed in grossly shaped patches positioned at the nuclear edge, whereas the residual relaxed chromatin is found mainly at the nuclear center. The plasma membrane presents numerous and well-shaped villi, and some figures resembling phagocytic vacuoles are visible. Numerous vesicles are detectable, some of which of large size, as well as electron-dense granules, especially detectable at the cell periphery. Notably, all the features described (segmented nuclei, chromatin condensed in patches, dilated perinuclear space, phagocyte vacuoles, and electrondense granules) are typical of circulating granulocytic cells: this is suggestive of granulocytic differentiation induced in U937 cells by the low etoposide dose: notably, physiological granulocyte differentiation is preceded by nuclear blebbing (Nusbaum et al., 2004) and DNA breaks (Weiss and Ito, 2015).

Figure 4B shows cells presenting both apoptosis and granulocyte-like characteristics. In these cells, the nuclei are apoptotic, the villi are absent, but many granules at the cell periphery or in the process of being extruded are detectable.

Overall, these images indicate that 0.5 uM etoposide promotes granulocytic-like differentiation in U937 cells, and that apoptosis may superimpose as an independent process.

### 0.5 uM Etoposide Promotes Intracellular Granularity and Oxidative Burst

We investigated 0.5 uM etoposide-induced putative granulocytic differentiation from the functional point of view, focusing on two characteristic features, namely granularity and oxidative burst, and their sensitivity to PMA challenge.

**FIGURE 5:**
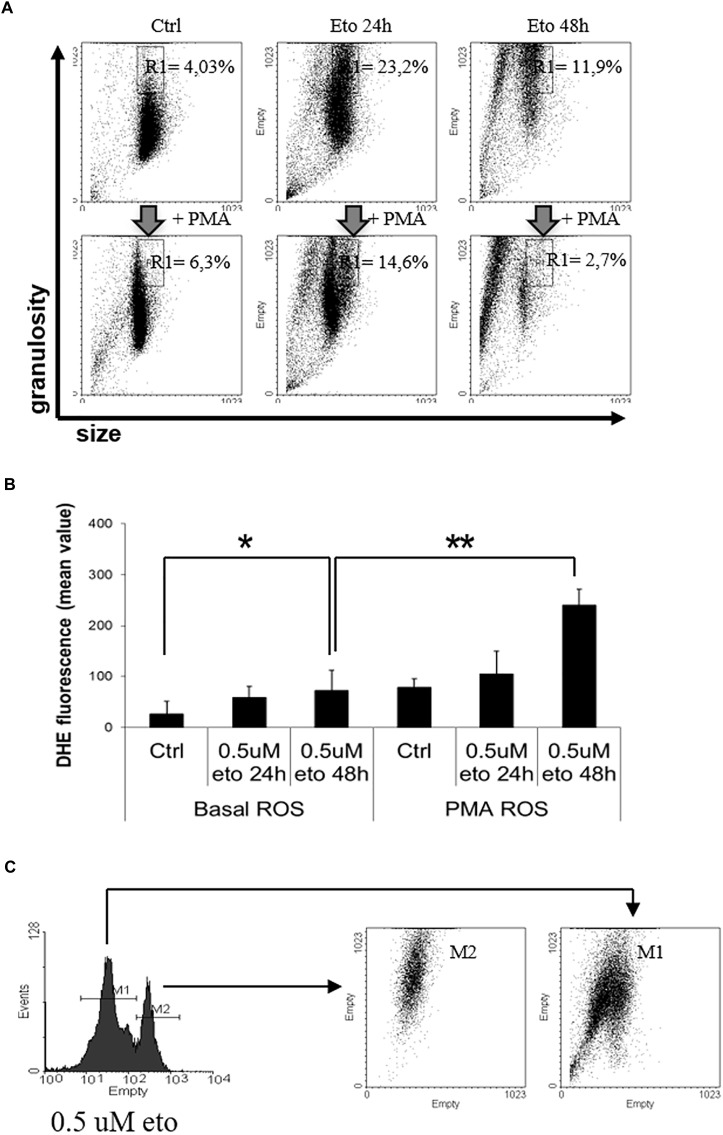
0.5 uM etoposide promotes intracellular granularity in U937 cells. **(A)** Cytofluorimetric dot plot analysis of forward (size) vs. side (granularity) scatter in U937 cells untreated or after 24 or 48 h of 0.5 uM etoposide, before (top line) or at 30 min after addition of 200 ng/mL PMA (bottom line). Representative histogram of 3 experiments performed with similar results; the % values indicate the fraction of cells falling in the gated area. **(B)** Cytofluorimetric analysis of superoxide production (DHE signal) in U937 untreated or after 24–48 h of 0.5 uM etoposide, before (left bars) or at 30 min after addition of 200 ng/mL PMA (right bars). Results are provided as the mean value, and are the average of 3 independent measurements ± SD: ^∗^*p* < 0.05 and ^∗∗^*p* < 0.01. **(C)** Cytofluorimetric profile of DHE stained U937 at 48 h after 0.5 uM etoposide (left); the right graph shows the side vs. forward scatter dot plot of the cells falling in the two DHE peaks.

Granularity is accompanied by intracellular dishomogeneity, detectable by flow cytometric analysis as an increase in side scatter; this is the principle at the basis of the flow cytometric detection of granulocytes in routine blood counts. Therefore, we explored whether 0.5 uM etoposide would produce an up-shift of cell side scatter. Figure 5A (top line) shows the side (granularity) vs. forward (size) scatter dot plot of U937 cells: 0.5 uM etoposide causes a strong increase in side scatter, as evident from the displacement of the cell population upward; this supports the morphological TEM analysis showing increased cell granulation. Interestingly, a second population of smaller cells (lower forward scatter) appears: it is tempting to speculate that such cells might be the result of size loss due to the extrusion of the large vesicles detected by TEM (see Figure 4B), in accordance with previous observations (MacGlashan, 1995).

Granulocytic differentiation implies also production of superoxides due to the transactivation of myeloperoxidases (Watanabe et al., 2001); Figure 5B (left panel) shows that 0.5 uM etoposide increases superoxide levels (especially at 48h, compare first with third bar), here measured as the increase in the mean fluorescence value of the specific probe dihydroethidium (DHE) signal. The profile of DHE-stained cells reveals that the increase is not homogeneous, but cells distribute in two separate populations with low ( = control level) and high DHE signal (Figure 5C, left). Importantly, flow cytometric bi-parametrical analysis shows that the cells belonging to the high DHE population (but not those with low DHE), also display high granularity (Figure 5C), showing that the two features are associated, occurring simultaneously in the same cells: this establishes a strong correlation, and reinforce the notion of induced granulocytic differentiation.

Phorbol myristate acetate (PMA) is a potent de-granulating agent in differentiated granulocytes (Parmley et al., 1983), and a strong activator of myeloperoxidase, abruptly promoting an oxidative burst (Collins et al., 1979); therefore, we used PMA to probe the nature of the 0.5 uM etoposide-induced increase granularity and superoxide production. Strikingly, PMA rapidly (30′) and strongly reverses the increase in side scatter induced by 0.5 uM etoposide without altering SSC in control cells (Figure 5A, compare top vs. bottom lines), thus behaving as a differentiation-specific de-granulating agent; notably, PMA pushes the majority of cells toward a reduced size: the swiftness of the response (30′) reinforces the hypothesis that de-granulation is associated to substantial volume loss due to massive macro-vesiculation. In addition, PMA also strongly increases the DHE signal on cells treated with 0.5 uM etoposide, but not on control cells (Figure 5B, compare the third vs. the sixth bar), showing also in this case that it behaves as a differentiation-specific inducer of oxidative burst.

All these evidences indicate that 0.5 uM etoposide induces granulocytic differentiation in U937 cells, and that high granularity may be used as a marker of differentiation in this system.

### 0.5 uM Etoposide Promotes a Caffeine-Sensitive DNA Damage Response, Which Determines Apoptosis and Differentiation

**FIGURE 6:**
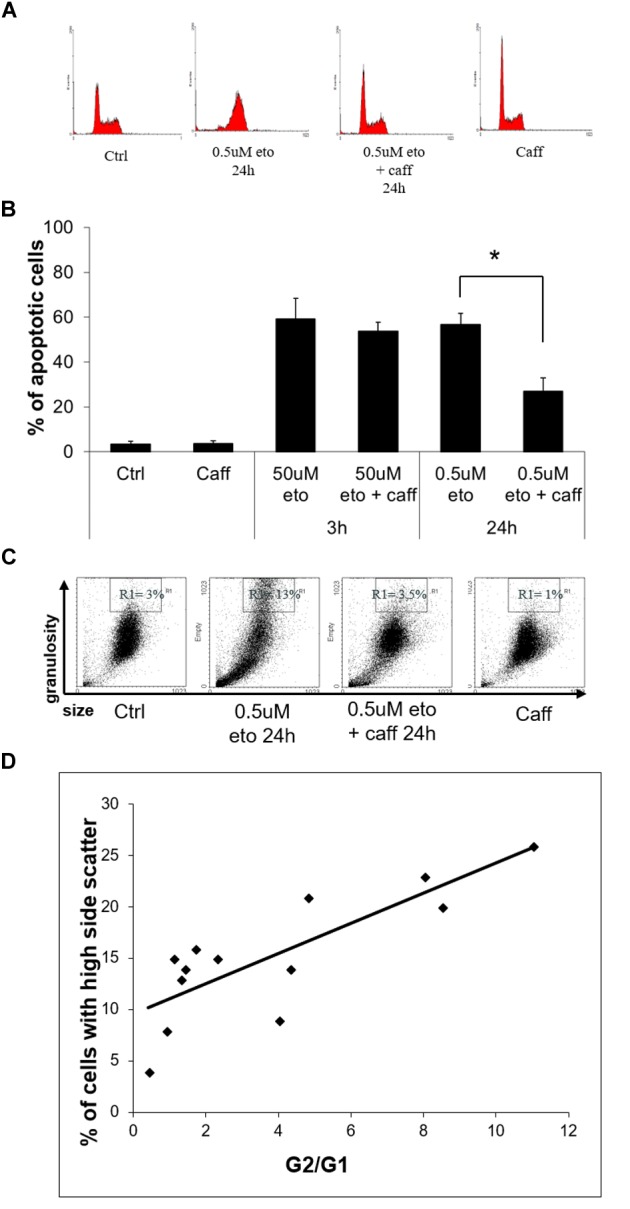
0.5 uM etoposide induces a caffeine-sensitive DNA damage response determining apoptosis and differentiation. **(A)** Cell cycle analysis of U937 cells treated with 0.5 uM etoposide ± caffeine (caff). Representative histograms of 4 experiments performed with similar results. **(B)** Apoptosis (nuclear fragmentation) in U937 cells treated with 50 uM or 0.5 uM etoposide ± caffeine (caff). Results are representative of 3 independent experiments ± SD: ^∗^*p* < 0.05. **(C)** Cytofluorimetric dot plot analysis of forward (size) vs. side (granularity) scatter in U937 cells untreated or after 24 h of 0.5 uM etoposide ± caffeine (caff). Representative dot plot of 3 experiments performed with similar results. **(D)** Correlation between G2 arrest and granulation induced by 0.5 uM etoposide at 24 h. G2/G1 ratio was calculated on the basis of quantification of cells in each phase of the cell cycle by means of specific markers (X-axis); the % fraction of cells with higher side scatter (SSC) was calculated as described for panel B (Y-axis). *R*^2^ = 0.6513 indicates good linear correlation.

DNA damage halts the cell cycle via the DNA damage response (DDR), causing alteration in the abundance of cells in the different phases (Caputo et al., 2012). At 24 h of 0.5 uM etoposide, apoptotic cells are detectable in the sub-G1 region, whereas viable cells change their distribution in the cycle with respect to control, enriching the G2 region (Figure 6A), as expected in cells undergoing DDR after DNA damage. The G2 cell cycle arrest occurs in an ataxia-telangectasia-mutated (ATM)-mediated fashion, since the ATM inhibitor caffeine (Dahl et al., 2005) prevents the arrest (Figure 6A).

We ascertained that caffeine did not just decrease etoposide activity, as sometimes reported (Pérez et al., 1994), by demonstrating that in our system caffeine did not reduce the extent of primary DNA breaks induced by 0.5 uM etoposide, as detected by alkaline comet assay (data not shown). With this information, we could use caffeine as a tool to investigate whether apoptosis and differentiation induced by 0.5 uM etoposide may be mediated by DDR, a process known to determine the fate of DNA damaged cells.

We observed that caffeine reduces the extent of 0.5 uM, but not 50 uM etoposide-induced apoptosis (Figure 6B): this indicates that the apoptosis induced by the low dose is not a direct effect of cytotoxicity, but the result of a signaling triggered by DNA damage response.

Importantly, caffeine also prevents the side scatter increase caused by 0.5 uM etoposide (Figure 6C), showing that also differentiation is DDR-mediated. In support to this conclusion, we report that the extent of 0.5 uM etoposide-induced SSC increase always correlates with the extent of cell accumulation in G2: Figure 6D shows the quantification of the G2 vs. G1 ratio at 24 h of 0.5 uM etoposide, plotted against the fraction of cells with high side scatter, separately measured in each of the experiment performed, pointing to a significant linear correlation between the two parameters.

All this evidence indicates a cause-effect relationship between the ATM response and the acquisition of both apoptosis and high granularity in U937 cells treated with 0.5 uM etoposide, showing that caspase-independent apoptosis and differentiation induced by 0.5 uM etoposide are mediated by the DNA damage response.

We found that the dual effect of the high vs. low doses of etoposide applies also to other myeloid leukemia cells such as KG1, THP-1 and HL-60, which display rapid, caffeine-independent, caspase-dependent apoptosis upon 50 uM etoposide treatment, and caffeine-sensitive caspase-3-independent apoptosis and caffeine-dependent increased granularity at 0.5 uM (data not shown).

## Discussion

A main message from this study is that a quantitative difference (dose of the same drug on the same cells), may induce different qualitative consequences, in this case, two different types of apoptosis. That cells can assess the extent of induced damage through “counting” mechanisms, is common knowledge: for example, we had reported in the past that the severity of the hyper-thermic or oxidative damage may shift the mode of cell death from apoptosis to necrosis (Ghibelli et al., 1992; Nosseri et al., 1994). Here, we report that the molecular mechanisms contributing to the decision between two types of etoposide-induced apoptosis, pass through the DNA damage response.

The importance of cell molecular response to damage, in addition to the mere consequence of it (i.e., death or survival), is beginning to be considered in anticancer therapeutic prospective. DDR actors such as Chk1 (Bartek and Lukas, 2003) or ATM (Shiloh, 2003) are emerging targets of novel radio-chemo-therapy adjuvant drugs for inhibiting DDR thus preventing DNA repair after therapy (Khoo et al., 2014; O’Connor, 2015). Aim is magnifying damage and increasing the apoptotic outcome of the treatment. This is based on the notion that DDR activates a powerful DNA repair machinery (Matt and Hofmann, 2016). A concern to the therapeutic use of this strategy comes from the consideration that mildly damaged cells may survive and replicate even in presence of non-repaired DNA damage: therefore, in such cases, repair inhibition may favor preserving any induced genetic alterations, thereby increasing risk of cancer development, and progression. Moreover, it must be considered that in addition to perform DNA repair, DDR is able to activate apoptosis, senescence (Sherman et al., 2011) and terminal differentiation (see below), thus promoting elimination of damaged, potentially dangerous cells: by inhibiting DDR, also such tumor preventing effects would be inhibited. In case of sub-lethal damage, apoptosis might be reached, by definition (sub-lethal = below threshold of lethality), only via signaling pathways, i.e., by a properly functioning DDR. In such instances, DDR inhibition may decrease, instead of increase the apoptotic outcome, since repair would not be the main issue. This is what we report here, showing that the DDR inhibitor caffeine decreases the extent of apoptosis induced by low etoposide dose (i.e., with mild damage), implying that the cells rescued by caffeine can die only thanks to DDR signaling: this underscores the role played by DDR in the genetic integrity surveillance processes. In fact, a vast literature reports dual, opposite effects of DDR inhibitors in cell survival to genotoxic stress (Inomata et al., 2009; Toledo et al., 2011; Roos and Kaina, 2013): it is tempting to interpret such discrepancies by hypothesizing that for light damage, DDR mainly acts by addressing sub-lethally damaged cells to apoptosis (pro-apoptotic action), whereas for strong damage, DDR would promote cell survival by reducing DNA damage (anti-apoptotic action). DDR is a pathway so heavily branched, to constitute a hub allowing many different, even opposite, end-points, depending on the inducer, and the microenvironment context. DDR is therefore a Janus-like controller, crucial in determining the response to cytotoxic chemo- or radio-therapies; it is highly likely that therapeutic success or failure depend on the sub-pathways put in motion by DDR.

The evidence that drug dosage can determine differential effects not only in quantitative, but also in qualitative terms, may be a key to approach the still not sharply defined, multifaceted mechanisms as basis for the therapeutic success of metronomic low-dose therapy. Metronomic low-dose chemotherapy is a clinically relevant therapeutic approach (Hart et al., 2015) characterized by the administration of regular cytotoxic drugs, i.e., DNA damaging agents or spindle poisons, with low, daily repeated doses, instead of the traditional maximum tolerable dose, delivered in 3- or 4-weekly pulses. Metronomic therapy implies better tolerability, anti-angiogenetic effect exploiting the hypersensitivity of endothelial cells to DNA damage-induced cell death (Bertolini et al., 2003; Rajasekaran et al., 2017), direct killing of cancer cells (Benzekry et al., 2015; Kareva et al., 2015), induction of tumor cell to senescence (Taschner-Mandl et al., 2016), immune infiltration regulation (Rozados et al., 2010; Wu and Waxman, 2014; Kareva et al., 2015; Biziota et al., 2017), stroma activity modulation (Chan et al., 2016), setting up efficient microenvironment reprogramming strategies against cancer (Hart et al., 2015).

The etoposide concentrations we used in our study conceivably represent cytotoxic (50 uM) vs. metronomic (0.5 uM) doses (Pasquier et al., 2013). Therefore, our results can be interpreted in this context; both concentrations lead to total cell apoptosis, achieved with different kinetics (few hours vs. 3 days), indicating that in this case dosage determines timing and type, rather than extent, of apoptosis. We had previously shown that slow release of etoposide from dextran conjugation also changes the type of apoptosis with respect to the nominal high concentration of 50 uM from cytotoxic- to differentiation-related (De Nicola et al., 2017), possibly constituting a useful device for stabilization of low concentrations also *in vivo* prospective usage. In both cases, the fact that different apoptotic pathways are elicited by different doses, suggests that the reprogramming effect metronomic approaches exert on cancer microenvironment, may include the ability to address DDR toward an anti-tumor behavior.

Repopulation is not an automatic response to cell loss, rather being the result of active signaling pathways. In particular, repopulation elicited by cells dying by caspase-3-dependent apoptosis, activating PGE2-mediated cell proliferation via the “phoenix rising” pathway (Li et al., 2010), is especially important since it causally associates cell loss with regeneration, providing to tissue repair and regeneration. However, not always apoptosis is followed by repopulation in physiological settings, rather aiming at eliminating surplus or dangerous cells. Interestingly, caspase-independent apoptosis physiologically occurs with higher frequency than previously noticed, often as an anticancer defense: notably, caspase-3-negative cell death occurs in anti-tumor instances such as spontaneous (Kitanaka et al., 2002) or immune-mediated (Ren et al., 2012) tumor regression, or EGF-induced differentiation (Fombonne et al., 2004). Clinically, high caspase-3 levels are a bad prognostic factor in cancer patients (Amptoulach et al., 2015), and caspase inhibition has been proposed as adjuvant to prevent repopulation (Flanagan et al., 2016): all this suggests that aiming at inducing caspase-3-independent apoptosis would be a sensible goal of anticancer therapies. We are presently setting up an *in vitro* system where to extend our analysis on caspase-3- dependent vs. independent apoptosis to other cell/drug systems, and mostly, to link the type of apoptosis to repopulation (Corsi et al., unpublished).

We have shown here that low etoposide doses act as pro-differentiation agents through the DNA damage response, suggesting a modulatory role of mild DNA damage. Interestingly, it is reported that DNA breaks play a role in precursor blood cell differentiation (Weiss and Ito, 2015) and granulocytic differentiation in bone marrow (Sjakste and Sjakste, 2007). U937 cells can be differentiated *in vitro* by modulation with DMSO, ATRA or PMA toward the monocytic, but not the granulocytic lineage (Zhang et al., 1992): however, we showed here that if DNA breaks are exogenously induced, e.g., by etoposide, then the avenue to granulocyte differentiation may re-open also for U937. This suggests that the signals from the DNA damage response may be more potent than the regular modulators, and once again reinforces the evidence of anticancer biomodulatory and reprogramming actions of low-dose chemotherapy, acting in fact as an anakoinosis inducer (André et al., 2018).

In conclusion, in therapeutic perspectives it is not only the extent of apoptosis induced on cancer cells that determines the therapeutic outcome of anticancer therapies, but also the cell response and the type of cell death must be taken into account, considering that the final therapeutic effects depend on the dynamics existing between induced apoptosis and tissue repopulation, in addition to the well-known systemic effects. Consequently, the response of cancer to cytotoxic drugs depends on complex reprogramming mechanisms controlled by tumor microenvironment, recently described in clinical and translational studies as anakoinosis, the communicative reprogramming anticancer therapy Anakoinosis (Hart et al., 2015), recently emerging as an innovative approach achieving important results even on metastatic cancers (Thomas et al., 2015; Hart et al., 2016; Ugocsai et al., 2016; Walter et al., 2017). Indeed, the findings that low dose anticancer agents may promote and control signaling pathways, provide evidence of a pro-anakoinosis effect of DNA damaging anticancer drugs, even including pro-differentiation effects implying cell reprogramming.

## Author Contributions

EmB made all the cell experiments and prepared the figures. ElB and MS performed the ultrastructural analysis and provided interpretation. AR and LG projected the study. AR provided the translational background. LG ideated the experiments and wrote the paper, which was discussed among all authors and approved.

## Conflict of Interest Statement

The authors declare that the research was conducted in the absence of any commercial or financial relationships that could be construed as a potential conflict of interest.
